# Isolation, Identification, and Biological Activity Analysis of Swim Bladder Polypeptides from *Acipenser schrencki*

**DOI:** 10.3390/foods12101934

**Published:** 2023-05-09

**Authors:** Xiao-Yan Zu, Wen-Bo Liu, Guang-Quan Xiong, Tao Liao, Hai-Lan Li

**Affiliations:** 1 Key Laboratory of Cold Chain Logistics Technology for Agro-Product (Ministry of Agriculture and Rural Affairs), Institute of Agro-Products Processing and Nuclear Agricultural Technology, Hubei Academy of Agricultural Sciences, Wuhan 430064, China; zuxiaoyan@hbaas.com (X.-Y.Z.);; 2 School of Chemical and Environmental Engineering, Wuhan Institute of Technology, Wuhan 430205, China

**Keywords:** *Acipenser schrencki*, swim bladder polypeptides, antioxidant peptides, peptide sequence

## Abstract

Swim bladder polypeptides (SBPs) of *Acipenser schrencki* were analyzed for their antioxidant activity and physicochemical properties. The results showed the optimal enzymatic conditions were alkaline protease with a solid-to-liquid ratio of 1:20, an incubation time of 4 h, a temperature of 55 °C, and an enzyme dosage of 5000 U/g. Three different molecular weight fractions (F1, F2, and F3) were obtained via ultrafiltration. F3 (912.44–2135.82 Da) showed 77.90%, 72.15%, and 66.25% removal of O_2_•^-^, DPPH•, and •OH, respectively, at 10 mg/mL, which was significantly higher than the F1 and F2 fractions (*p* < 0.05). F3 contained proline (6.17%), hydroxyproline (5.28%), and hydrophobic amino acids (51.39%). The UV spectrum of F3 showed maximum absorption at 224 nm. Peptide sequence analysis showed that F3 contained antioxidant peptides (MFGF, GPPGPRGPPGL, and GPGPSGERGPPGPM) and exhibited inhibitory activities on angiotensin-converting enzyme and dipeptidyl peptidase III/IV (FRF, FPFL and LPGLF). F3 was considered a good raw material for obtaining bioactive peptides.

## 1. Introduction

Reactive oxygen species (ROS), for example, superoxide anion radicals (O_2_•^-^), hydroxyl radicals (•OH), and hydrogen peroxide (H_2_O_2_), are indispensable cellular components in living organisms that are involved in the regulation of cellular signal transduction and the cell cycle. However, excess ROS is almost always linked to cancer, aging, or other diseases. Furthermore, oxidation during food processing and storage may lead to food spoilage [1]. The nutritional value of meat is decreased by oxidation as a result of the loss of vitamins and essential fatty acids. The initial alteration observed causes the quality of the experience to gradually decline. For instance, changes in color and texture and even foul smells can affect consumer acceptance [2]. Additionally, by causing inflammation, oxidative stress plays a crucial part in the genesis of several chronic diseases, including diabetes and cancer [3]. In recent years, it has been demonstrated that peptides from traditionally inedible or non-traditional foods function as exogenous antioxidants and improve the antioxidant defense system of the body or act as food additives to inhibit the oxidative deterioration of foods [4]. Moreover, due to their nutritional value and safety, these peptides have been suggested as suitable substitutes for chemical antioxidants [5]. Therefore, extensive investigations have been conducted on antioxidant bioactive peptides from various sources.

Vertebrate skin, bone, and connective tissue contain abundant collagen, which has great potential to produce bioactive peptides through enzymatic hydrolysis. The bioavailability and safety of collagen peptides obtained from fish are superior to those derived from terrestrial animals. Moreover, they are accepted by various religious groups [6]. According to reports, collagen peptides with favorable antioxidant properties may be obtained from fish skin [7], fish scales [8] and fish bones [9]. Active components with DPPH•, •OH, and O_2_•^-^ scavenging activities have been obtained after enzymolysis and separation of the collagen obtained from the swim bladders of the giant croaker (*Nibea japonica*) and grass carp (*Ctenopharyngodon idella*) [10,11]. Furthermore, it has been shown that collagen peptides obtained from the swim bladders of the gulf corvina (*Cynoscion othonopterus*) and the bighead carp (*Hypophthalmichthys nobilis*) were more thermally stable than those extracted from fish skin and fish bone [12,13]. However, to date, there have been few reports on the properties of collagen and its enzymatic products from the swim bladders of sturgeons.

*Acipenser schrencki* is a riverine resident sturgeon species found in the Amur (Heilongjiang) River, which accounts for almost 15% of the total production of Chinese farmed sturgeon [14]. Sturgeon farming has been the fastest-growing area of aquaculture since the turn of the 20th century due to the huge demand for caviar [15]. According to the latest Fishery and Aquaculture Statistics published by the Food and Agriculture Organization (FAO), the aquaculture production of sturgeons reached 120,000 tons in 2019 [16]. The sturgeon swim bladder is often discarded as a byproduct during processing. The inadequate utilization of a large amount of swim bladders results in a huge waste of resources as well as environmental pollution [17].

In this study, the enzymatic hydrolysis conditions of the swim bladders of sturgeons were optimized, and ultrafiltration was performed to purify and separate swim bladder polypeptides (SBPs) with different molecular weights. Free radical scavenging ability and Fe^3+^ reducing power were evaluated on the various peptide fractions that were produced. Target peptides were subject to analysis of their amino acids (AAs), characteristic absorption peaks, molecular weights, and peptide sequences using matrix-assisted laser desorption/ionization time-of-flight mass spectrometry (MALDI-TOF-MS, Bruker Ultraflextreme, Bremen, Germany) and liquid chromatography with tandem mass spectrometry (LC-MS/MS, Ultimate 3000 UHPLC-Q Exactive, Thermo Fisher, Boston, MA, USA) to provide support for the potential applications of SBPs in the food industry.

## 2. Materials and Methods

### 2.1. Raw Materials and Reagents

A sample swim bladder of *Acipenser schrencki* weighing 1000 g (frozen) was supplied by Hubei Qingjiang Sturgeon Valley Special Fisheries Co. (Yichang, China). Alkaline protease (200 U/L) was provided by Wuxi UPG Bio-technology Co., Ltd. (Nanjing, China). Sinopharm Chemical Reagent Co., Ltd. (Shanghai, China) supplied papain (6000 U/mg). Neutral protease (50 U/mg), nitro blue tetrazolium (NBT), b-nicotinamide adenine dinucleotide (NADH), 2,2-diphenyl-1-picrylhydrazyl (DPPH), potassium ferrocyanide, phenazine methosulfate (PMS), and trifluoroacetic acid (TFA) were provided by Shanghai Macklin Biochemical Technology Co., Ltd. (Shanghai, China). All reagents and chemicals were of analytical grade.

### 2.2. Determination of Basic Content

The chemical components of the sturgeon swim bladder were tested using the AOAC method [18]. The crude protein content was determined by the Kjeldahl method (*n* = 6.25). The residue-on-evaporation method (105 °C) was used to measure the moisture content of the swim bladder. By using petroleum ether and reflux extraction, fat content was ascertained. A muffle furnace operating at 550 °C was used to measure the total ash content. All experimental samples were prepared in triplicates. An atomic absorption spectrophotometer (iCE 3500, Thermo Scientific, Boston, MA, USA) was used to determine the mineral composition. The contents (mg/100 g) of potassium, magnesium, calcium, iron, and zinc were about 50.1, 20.4, 21.5, 1.3, and 3.5, respectively.

### 2.3. Enzymatic Digestion and Ultrafiltration

The raw materials were cleaned, cutting them into around 0.5 cm × 0.5 cm pieces. To remove fat, the pieces were immersed in 0.5% NaHCO_3_ (1:20 *w/v*) for 8 h and 0.6 M NaCO_3_ (1:15 *w/v*) for 6 h (repeated twice), repeatedly rinsed to ensure neutrality, dried, and refrigerated for subsequent use. Papain, alkaline protease, and neutral protease were used for enzymatic hydrolysis. The enzymes were then inactivated in a boiling water bath for 20 min at 100 °C. The product was centrifuged in a centrifuge (TGL-24MC, Changsha Pingfan Instrument Co., Ltd., Hunan, China) at 18 °C and 7155× *g* for 20 min. The supernatant was collected to adjust the pH to neutrality, followed by freeze-drying to obtain a light-yellow SBP powder. The DPPH• scavenging activity and degree of hydrolysis (DH) were used as indicators to assess the enzymatic effect and determine the optimal enzyme.

Single-factor tests were used to identify the ideal conditions for the enzymatic hydrolysis of the swim bladder of sturgeons. The impacts of the solid-to-liquid ratio, enzymatic digestion duration, temperature, and enzyme amount on DH and DPPH• scavenging activity were investigated sequentially (Table 1). The SBPs (10 mg/mL) obtained with the optimal enzymatic conditions were ultrafiltered using a laboratory nanofiltration membrane system (LNG-NF-101, Shanghai Laungy Membrane Separation Equipment Engineering Co., Ltd.). The SBPs were ultrafiltered using ultrafiltration membranes with intercepted molecular weights of 10, 5, and 3 kDa (polyethersulfone resin with a filtration area of 0.24 m^2^) in turn to obtain three fractions, namely F1 (Mw: 5 –10 kDa), F2 (Mw: 3 –5 kDa), and F3 (Mw: 0 –3 kDa), respectively. The product was vacuum freeze-dried for subsequent use.

### 2.4. Determination of Degree of Hydrolysis (DH)

The pH-stat method was used to determine the DH [19]. During enzymatic hydrolysis, 0.1 mol/L NaOH was used to maintain constant pH, and the volume of NaOH consumed (mL) was recorded. The calculation formula is as follows:

DH = (h/h_tot_) × 100% = [(V × N_b_)/(α × M_p_ × h_tot_)] × 100%

where h is the hydrolyzed peptide bond number, h_tot_ is the peptide bond number in the protein substrate (mmol/g_protein_), V is the NaOH consumed (mL) volume, N_b_ is the NaOH (mol/L) concentration, α is the average dissociation degree of amino groups, and M_P_ is the total amount of protein in the substrate (g).

### 2.5. Antioxidant Assay

#### 2.5.1. DPPH• Scavenging Activity

Teng et al., (2011) were referred to for the measurement of DPPH scavenging activity [20]. A total of 2 mL of samples (2, 4, 6, 8, 10 mg/mL) and 2 mL of 65 μM DPPH-ethanol solution were mixed and kept away from light for 30 min. Using a UV-Vis spectrophotometer (UH5300, Hitachi, Ltd., Tokyo, Japan), the absorbance (A) was determined at 517 nm. The calculation formula was as follows:

DPPH clearance rate (%) = [1 − (A_1_ − A_2_)/A_0_] × 100%

where A_1_ is the A-value of 2 mL sample + 2 mL 65 μM DPPH, A_2_ is the A-value of 2 mL 95% ethanol + 2 mL sample, and A_0_ is the A-value of 2 mL DPPH + 2 mL 95% ethanol.

#### 2.5.2. O_2_•^-^ Scavenging Activity

The method of Wang et al., (2008) was utilized as a reference to assess the O_2_•^-^ scavenging capacity of the samples [21]. O_2_•^-^ was generated in 2.8 mL of Tris-HCl buffer (50 mM, pH 8.0), which contained 0.25 mL of NBT (300 mM), 0.25 mL of NADH (468 mM) solution, and 100 μL of the sample (2, 4, 6, 8 and 10 mg/mL). Then, 0.25 mL of PMS (60 mM) solution was added to the mixture to react for 5 min at room temperature. The A-value at 560 nm was determined using a spectrophotometer. The formula was as follows.

O_2_•^-^ scavenging activity (%) = [(A_0_ − A_1_)/A_0_] × 100%

where A_0_ refers to the blank group and A_1_ refers to the sample group.

#### 2.5.3. •OH Scavenging Activity

The method of Chen et al., (2019) was slightly modified and used as a reference [10]. Sample solutions with 2, 4, 6, 8, and 10 mg/mL concentrations were prepared. A 10 mL centrifuge tube was filled with 2 mL of sample solution, followed by consecutive additions of 1 mL of ferrous sulfate (9 mM), salicylic acid–ethanol (9 mM), and hydrogen peroxide (8.8 mM) solutions. Following 30 min of reaction in a water bath at 37 °C, the A-values of the solutions were measured at 510 nm. The calculation formula was as follows:

•OH scavenging activity (%) = [A_0_ − (A_1_ − A_2_)]/A_0_ × 100

where A_0_ is the absorbance of the solution without the sample, A_1_ is the absorbance of the sample, and A_2_ is the absorbance of the sample without hydrogen peroxide.

#### 2.5.4. Fe^3+^ Reducing Power

The reducing power of SBPs was determined based on the method by Xia et al. [22]. The sample (2, 4, 6, 8, and 10 mg/mL) was mixed thoroughly with phosphate buffer (0.2 M, pH 6.6) and potassium ferricyanide solution (10%) and incubated at 50 °C for 20 min. The mixture was then centrifuged at 25 °C and 5000× *g* for 10 min after the trichloroacetic acid solution (10%) was added. The supernatant and deionized water were obtained to react with FeCl_3_ (0.1 M) for 10 min. The A-value of the solution was measured at 700 nm, and no sample was added to the blank group. A greater A-value was associated with a higher reducing power of the sample.

### 2.6. Analysis of Target Peptides

#### 2.6.1. AA Composition

Following the procedure described by Teng et al., (2011) [20], samples were hydrolyzed with HCl (6 M) in a vacuum for 24 h at 110 °C and evaluated using an automated AA analyzer (L8900, Hitachi, Ltd., Tokyo, Japan). The main assay conditions were a sulfonic acid cation-exchange resin of 2.6 mm × 150 mm, a detection wavelength for proline of 440 nm, and a detection wavelength for other AAs of 570 nm. The sample detections were performed in triplicate.

#### 2.6.2. UV Scanning Spectrum

The sample was dissolved in phosphate buffer at pH 7.0, and a UH5300 UV spectrophotometer (Hitachi, Tokyo, Japan) was used to determine the UV absorption peaks of each component at room temperature. The wavelength range was set to 190–400 nm.

#### 2.6.3. Molecular Weight Distribution

Samples were passed through a 0.25 μm membrane filter (Millipore, Billerica, MA, USA). Products were then mixed with an equal volume of α-cyano-4-hydroxycinnamic acid (CHCA) substrate solution. Then, 1 Μl of the mixture was loaded on a slide (MP384 non-polished steel plate, Bruker Daltonics) and allowed to dry naturally. Mass spectra were collected on a Bruker Daltonics Ultraflextreme MALDI-TOF mass spectrometer operating in linear positive mode. The analyzer was operated at an acceleration voltage of +20 Kv. An 8.02 Kv lens was utilized to concentrate the laser light on the sample. The pulsed ion extraction was optimized to 170 ns.

#### 2.6.4. Peptide Sequence Identification

The AA sequences were analyzed by LC-MS/MS. Solution A in the liquid phase was an aqueous solution of formic acid (0.1%), and solution B was an aqueous solution of formic acid–acetonitrile (0.1%, containing 84% acetonitrile). The RP-C18 liquid chromatographic column (0.15 mm × 150 mm, Column Technology Inc., Fremont, CA, USA) was equilibrated with 95% A solution. Additionally, the sample was injected via an autosampler into Zorbax 300SB-C18 peptide traps (Agilent Technologies, Wilmington, DE, USA) for separation on a liquid chromatography column. The liquid phase gradient was set as follows. From 0 min to 50 min, the linear gradient of solution B increased from 4% to 50%. From 50 min to 54 min, the linear gradient of solution B increased from 50% to 100%. From 54 min to 60 min, solution B was maintained at 100%. Mass spectrometry was analyzed using a Q-Exactive mass spectrometer (Thermo Fisher, Boston, MA, USA) in positive ion mode for 60 min. The mass spectrometry data were analyzed by MaxQuant 1.5.5.1, and the UniProt database search was set as follows: MS/MS tolerance was 0.2 Da with up to two missing fragmentations, and the methionine oxidation served as variable modifications. The confidence level for positive protein identification was determined based on the high protein and peptide fractions in the search results.

### 2.7. Statistical Analysis

Origin 2021 (Origin Lab Co., Ltd., Northampton, MA, USA) was used to plot the data. Student’s *t*-test was used to test for statistically significant intergroup differences (*p* < 0.05). Data results were expressed as mean ± standard deviation, and all measurements were repeated trebly (*n* = 3).

## 3. Results and Discussions

### 3.1. Basic Components of Swim Bladders

As shown in Table 2, sturgeon swim bladders are characterized by low-fat content and high amounts of protein. The high protein content seemed to be a common characteristic of swim bladders [23]. Collagen, which is abundant in fish swim bladders, is an intrinsic water-binding protein with moisturizing properties [24]. Zhao et al., reported that the water contents of swim bladders of the large yellow croaker and grouper were approximately 14.1–18.6%, with favorable moisture retention properties [25].

### 3.2. Optimization of the Enzymatic Process of Fish Bladder

**Figure 1:**
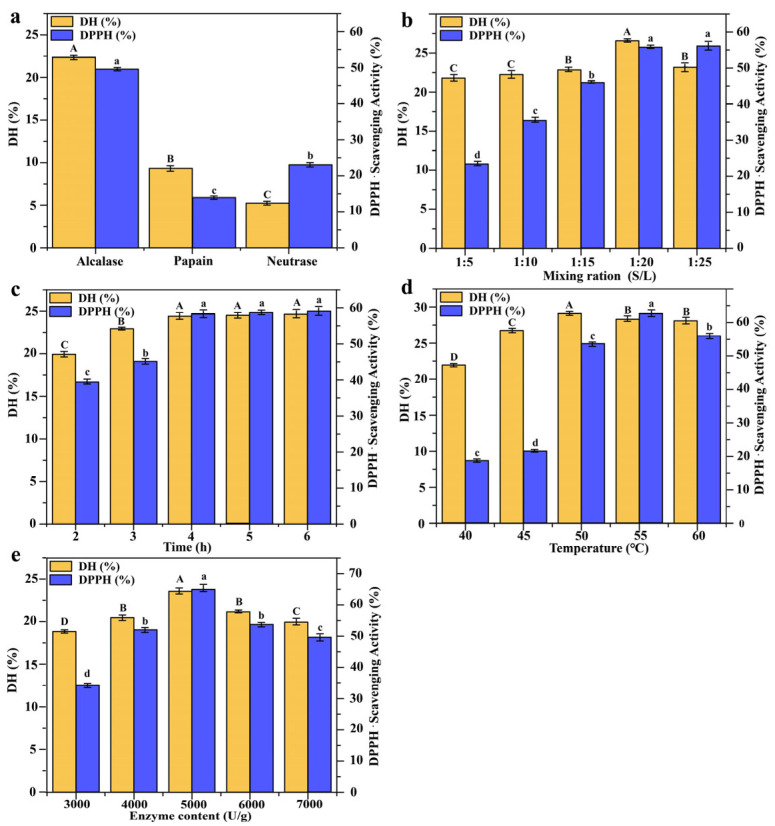
Effects of enzymatic hydrolysis conditions on DH and DPPH• scavenging ability. (**a**) Screening of enzymes; (**b**) solid-to-liquid ratio; (**c**) time of incubation; (**d**) temperature; (**e**) enzyme/substrate. Different uppercase and lowercase letters express that there are significant differences between different groups under the same evaluation index (*p* < 0.05).

As shown in Figure 1a, alkaline protease exhibited the optimal enzymatic hydrolysis with a DH up to 22.65% and a DPPH• scavenging rate of the obtained SBPs up to 49.88% (*p* < 0.05). In the process optimization experiments, the DPPH• scavenging rate of SBPs gradually increased to 55.85% with the increase in the solid-to-liquid ratio. The maximum DH was 26.57% at a solid-to-liquid ratio of 1:20. No significant change in DPPH• scavenging activity was observed when the solid-to-liquid ratio increased to 1:25. In contrast, DH was found to significantly decrease (*p* < 0.05) (Figure 1b). The DPPH• scavenging activity and DH of SBPs increased with the increase in enzymatic hydrolysis duration (Figure 1c) but were constant after 4 h. Temperature had a significant influence on the DPPH• scavenging activity of SBPs. At 40–60 °C, the DPPH• scavenging activity increased, but from then on, it declined with increasing temperature, with the DPPH• scavenging activity of SBPs reaching 62.67% at 55 °C (Figure 1d). The DPPH• scavenging activity and DH increased before decreasing according to increases in the amount of enzyme (Figure 1e), and the DPPH• scavenging activity of SBPs reached up to 64.59% at an enzyme amount of 5000 U/g. In contrast, excessive enzyme concentrations may result in aggregation that could inhibit substrate diffusion, thus causing a decrease in the reaction rate [26]. Therefore, the optimal reaction conditions for the enzymatic digestion of sturgeon swim bladders by alkaline protease were a solid-to-liquid ratio of 1:20 at 55 °C for 4 h with an enzyme amount of 5000 U/g.

### 3.3. Antioxidant Properties of Peptides with Different Molecular Weights

**Figure 2:**
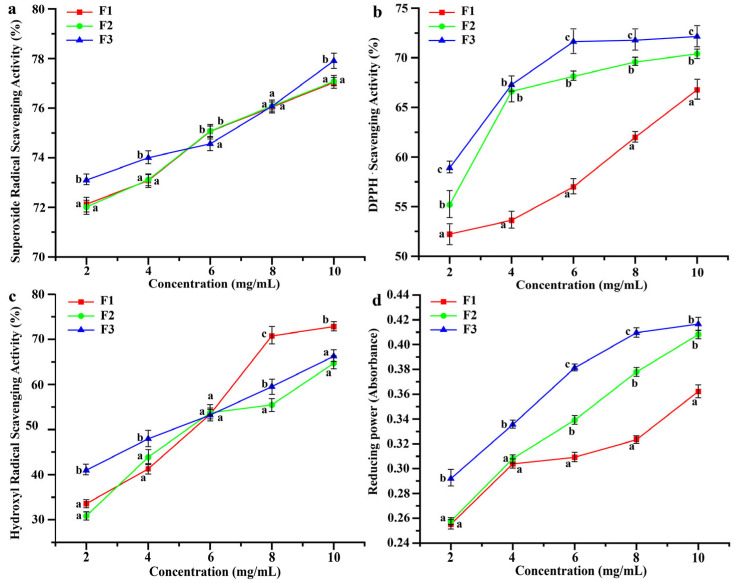
Antioxidant activity of F1, F2, and F3 (10.0 mg/mL). (**a**) Superoxide radical (O_2_•^-^) scavenging activity; (**b**) DPPH• scavenging activity; (**c**) hydroxyl radical (•OH) scavenging activity; (**d**) reducing power. Different lowercase letters indicate significant differences between different samples at the same concentration (*p* < 0.05).

Three different molecular weight fractions (F1, F2, and F3) were produced via ultrafiltration for antioxidant assays. Figure 2 shows that the scavenging activities of O_2_•^-^, DPPH•, and •OH, as well as the Fe^3+^ reducing the power of the three fractions, increased in a dose-dependent manner. At a concentration of 10 mg/mL, the scavenging activity of F3 for O_2_•^-^ (77.01%) was significantly higher than that of F1 and F2 (*p* < 0.05) (Figure 2a). DPPH• scavenging capacity increased with a decrease in the molecular weight of the fractions. Among them, the DPPH• scavenging rate of F3 reached as high as 72.15% at a concentration of 10 mg/mL (Figure 2b), which was consistent with the finding of Zhao et al., who enzymatically digested the swim bladder of miiuy croaker and obtained the best DPPH• removal by fractions less than 3 kDa [27].

All three fractions could scavenge •OH (Figure 2c). At a concentration of 10 mg/mL, the •OH scavenging activity of F1 (72.81%) was superior to that of F3 (*p* < 0.05). F3 had the greatest power to reduce Fe^3+^ to Fe^2+^ at the same concentration (Figure 2d). •OH is the most active free radical; it can induce lipid peroxidation in biological membranes and may initiate carcinogenesis, mutagenesis and cytotoxicity by reacting with biological macromolecules [28]. Reducing power plays an important role in assessing the antioxidant activity of peptides in reducing Fe^3+^ to Fe^2+^ [29]. There have been some reports that peptides with greater molecular weight exhibited a higher free radical scavenging capacity. For example, Chen et al., found that a fraction of soy protein with a molecular weight greater than 30 kDa exhibited higher DPPH scavenging activity [30]. In this experiment, the •OH scavenging activity of F1, the fraction with large molecules, was superior to that of F3. However, low-molecular-weight peptides are generally considered better antioxidants [31], which is consistent with the overall results for antioxidants in this study.

### 3.4. Analysis and Characterization of F3

#### 3.4.1. AA Composition of F3

As shown in Table 3, a total of 17 AAs were identified in F3, with glycine and glutamic acid contents being the highest and the second highest, respectively, along with high levels of proline and hydroxyproline. The hydrophobic AA content accounted for more than 50% of F3, with over 30% being essential and semi-essential AAs. Meanwhile, low levels of tyrosine and histidine were observed, which was consistent with the research results of grass carp and bighead carp swim bladders [11]. The swim bladder is rich in collagen, with glycine as the main AA and proline and hydroxyproline as the characteristic AAs [32]. Glycine is an AA that contributes to antioxidant activity, with the hydrogen atoms on the side chains quenching unpaired electrons as well as free radicals while allowing more functional groups in the peptide chain to become targets of free radicals [33]. Glutamic acid is susceptible to oxidative dehydrogenation to neutralize free radicals and therefore contributes to antioxidant activity [34]. In addition, the hydrophobic AAs, such as Phe at the N-terminal, facilitate scavenging free radicals, and peptides containing a high proportion of hydrophobic AAs usually have higher antioxidant activity [35]. These findings indicated that F3 was rich in AAs and also had a favorable potential for antioxidant activity.

#### 3.4.2. UV Spectrum of F3

**Figure 3:**
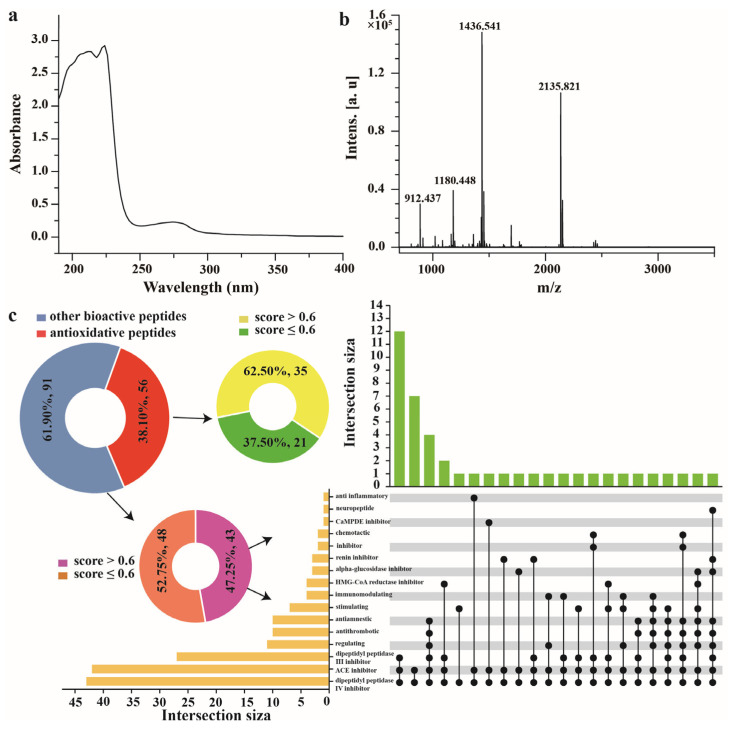
UV absorption spectrum (**a**), molecular weight (**b**), and potential biological activity analysis of F3 (**c**).

As shown in Figure 3a, the maximum absorption peak was observed at 224 nm, and a weak absorption peak was found at 250–280 nm on the UV spectrum of F3. The polypeptide chains of collagen contain carbonyl, carboxyl, and amide groups, which usually produce obvious absorption peaks at 210–240 nm. In this study, the absorption peaks of F3 were typical for collagen. Aromatic AAs would produce obvious absorption peaks at 250–280, for instance, phenylalanine and tyrosine [36]. Aromatic AAs were present in F3 but at a very low level, which was consistent with the AA composition analysis results in Table 3. Dong et al., found that the collagen of the swim bladders of four species of fish, including grass carp and bighead carp, had a maximum absorption peak at 230 nm [11], which was slightly different from the findings of this study and was probably due to the differences in AA sequence and protein composition.

#### 3.4.3. Molecular Weight of F3

As shown in Figure 3b, the molecular weight range of F3 was 912.43–2135.82 Da, with the two main molecular weight peaks being 1436.54 Da and 2135.82 Da. The AA sequence, composition, molecular weight, and hydrophobicity of peptides are all intimately connected to their antioxidant activity [37]. Peptides with low molecular weight within 1000–3000 Da have been considered to interact more easily and efficiently with free radicals [38]. Combined with the sequencing results, there were two peptides with molecular weights of approximately 1400 Da, namely GPRGPSGERGEVGPA (1421.701 Da) and KLPELPITNFSR (1413.798 Da). There were two peptides, namely AGDDAPRAVFPSIVGRPRHQ (2145.119 Da) and AVTVMIGGEPYTLGLFDTAGQ (2139.055 Da), whose N-terminals were hydrophobic AAs (G and A), with E, D, R, and K present in the sequence. The presence of hydrophobic AAs at the N-terminal is one of the characteristics of antioxidant peptides, and the presence of charged AAs may also contribute to the antioxidant activity of peptides [4,39].

#### 3.4.4. Peptide Sequence of F3

A total of 746 peptides were identified through database alignment (https://www.uniprot.org/) (accessed on 10 January 2023). The peptides ranged from 3 to 22 AA residues, i.e., their molecular weights were between 363 and 2400 Da, which was consistent with the molecular weight results in Figure 3b. Among them, 147 peptides had alignment scores >60. These sequences were submitted to the BIOPEP database (https://biochemia.uwm.edu.pl/) (accessed on 6 February 2023) for bioactivity prediction. All of these peptides contained known bioactive sequences, and the results showed that 56 of them demonstrated antioxidant activity, while another 91 peptides had the potential for ACE inhibitory activity and dipeptidyl peptidase III/IV inhibitory activity (Figure 3c).

PeptideRanker (http://bioware.ucd.ie/) (accessed on 8 February 2023) was used to rank the bioactivity potentials, and higher scores within 0–1 indicated greater bioactivity. Only peptides with scores above 0.6 were considered to be potentially biologically active. Twenty-one peptides with potential antioxidant activity were obtained (Table 4). Among them, the scores of MFGF, GPPGPRGPPGL, and GPGPSGERGPPGPM were 0.9943, 0. 9560, and 0.9038, respectively. Therefore, it was suggested that they might act as novel antioxidant peptides. MFGF, LLPL (0.7150), and LVFL (0.643) were also found to share the C-terminal sequences with reported antioxidant peptides [40,41]. Therefore, they may also have antioxidant potential.

In addition, LPGLF (with a score of 0.9486) exhibited seven biological activities, including ACE inhibitory activity and dipeptidyl peptidase IV inhibitory activity. Phenylalanine (F), leucine (L), and proline (P) were often found at the C-terminus of ACE inhibitory peptides, and the hydrophobicity of the AAs at the C-terminus was positively correlated with the ACE inhibitory activity [42]. Analysis of peptides isolated from gelatin hydrolysate of squid by A. Alemán et al., indicated that the presence of leucine played a crucial role in the ACE inhibitory activity [43]. Therefore, it is possible that LPGLF had good ACE inhibitory activity.

## 4. Conclusions

In this study, SBPs were obtained from *Acipenser schrencki* swim bladders by digestion under optimal conditions (alkaline protease, solid-to-liquid ratio 1:20, enzyme amount of 5000 U/g at 55 °C for 4 h). Three fractions, namely F1, F2, and F3, were obtained by ultrafiltration in descending order of molecular weight. Among them, F3 (Mw, 912.43–2135.82 Da) exhibited favorable scavenging activity of O_2_•^-^ and DPPH• as well as Fe^3+^ reducing power. F3 had a maximum absorption peak at 224 nm and was rich in glycine. Moreover, it contained the characteristic AAs proline and hydroxyproline, which were consistent with the characteristics of collagen peptides. A total of 56 peptide sequences with potential antioxidant activities were identified in F3 by LC-MS/MS, among which MFGF, GPPGPRGPPGL, and GPGPSGERGPPGPM ranked high in antioxidant activity. In addition, F3 also had potentially ACE-inhibitory peptide and dipeptidyl peptidase III/IV inhibitory peptide activities. In subsequent studies, cellular experiments will be conducted to verify the above biological activities and mechanism of action of F3.

## Figures and Tables

**Table 1:** Enzymatic hydrolysis process parameters and levels.

Solid-Liquid Ratio (g:mL)	Time (h)	Temperature (°C)	Enzyme/Substrate(U/g)
1:5	2	40	3000
1:10	3	45	4000
1:15	4	50	5000
1:20	5	55	6000
1:25	6	60	7000

**Table 2:** Approximate composition (%) of swim bladder from *A. schrencki*.

Components	Protein	Moisture	Ash	Fat
(%)	78.61 ± 4.30	15.17 ± 0.03	0.99 ± 0.02	2.97 ± 0.07

**Table 3:** AA composition of F3.

AA (%)	F3
HydroPro	5.28 ± 0.17
Asp	7.18 ± 0.08
Thr	3.56 ± 0.13
Ser	5.52 ± 0.24
Glu	12.21 ± 0.06
Pro	6.17 ± 0.15
Gly	21.57 ± 0.16
Ala	10.42 ± 0.07
Val	2.79 ± 0.11
Met	1.57 ± 0.03
Ile	2.29 ± 0.04
Leu	3.96 ± 0.12
Tyr	1.64 ± 0.07
Phe	2.62 ± 0.03
Lys	3.82 ± 0.02
His	1.29 ± 0.01
Arg	8.12 ± 0.05
HAA	51.39 ± 0.29
EAA	30.02 ± 0.15

HAA, hydrophobic AAs (Gly, Ala, Pro, Val, Leu, Ile, Phe, Met); EAA, essential AAs (Lys, Phe, Met, Thr, Ile, Leu, Val, Arg, His). F3 is the component with a molecular weight of less than 3 kDa obtained by ultrafiltration.

**Table 4:** Bioactive peptides predicted by PeptideRanker and BIOPEP.

Peptide Sequence	Protein ID.	Score	Peptide Ranker ^a^	Potential Bioactive Peptides ^b^	Biological Functions ^b^
MFGF	A0A444U3D5	65.81	0.9943	FGF	Antioxidative
GPPGPRGPPGL	A0A444U5J5	99.37	0.9560	GPP	Antioxidative
GPGGPSGERGPPGPM	A0A444TZY1	128.81	0.9038	GPP	Antioxidative
FDRPSPPPWAA	A0A444V784	107.15	0.8992	PWA, PW	Antioxidative
SGPPGFPGSPGPKGE	A0A662YXI2	81.33	0.8845	GPP	Antioxidative
GLPGPIGPPGPR	A0A444TZY1	70.85	0.8811	GPP	Antioxidative
FGGRPIPGSPF	A0A444TZH1	135.83	0.8642	GGRP	Antioxidative
GPRGPPGEPGL	A0A662YUK1	101.25	0.8567	GPP	Antioxidative
AVPGPPGEPGRL	A0A444UE44	68.22	0.8412	GPP	Antioxidative
GPPGKDGQPGHPGPIGPA	A0A0S3P5T6	179.59	0.8358	KD, GPP	Antioxidative
LPLL	A0A444UQF3	62.26	0.7150	LPL	Antioxidative
PGIPGPEGPR	A0A662YTX1	138.26	0.7121	GPE	Antioxidative
GIGPEGPHLGIV	A0A444V058	143.11	0.6989	HL	Antioxidative
AGDDAPRAVFPSIVGRPR	A0A444V306	172.93	0.6843	AGDDAPR	Antioxidative
SLYPPSEKPIMK	A0A444UV48	81.625	0.6822	LY, KP	Antioxidative
LLPL	A0A444UQC2	62.263	0.6800	LPL	Antioxidative
DVVDFPRFPHR	A0A444UZ64	100.48	0.6657	PHR	Antioxidative
GFAGDDAPRAVFPSIVGRPR	A0A444V306	130.27	0.6456	AGDDAPR	Antioxidative
LVFL	A0A444UQC2	69.29	0.6430	VFL	Antioxidative
VFLR	A0A662Z298	70.86	0.6161	VFL	Antioxidative
GIGPEGPHLGIVQ	A0A444V058	102.01	0.6093	HL, PHL, GPE	Antioxidative
FRF	A0A444UQC2	62.74	0.9954	RF, FR	ACE inhibitor
				FR	Dipeptidyl peptidase IV inhibitor
				RF, FR	Dipeptidyl peptidase III inhibitor
FPFL	A0A662YSN4	60.00	0.9930	FP	ACE inhibitor
				FP, FL, PF	Dipeptidyl peptidase IV inhibitor
				FL, PF	Dipeptidyl peptidase III inhibitor
FGLF	A0A444UCV7	83.87	0.9903	LF, GL, FG	ACE inhibitor
				GL	Dipeptidyl peptidase IV inhibitor
				GLF	Immunomodulating
				GLF	Regulation
FPAF	A0A444UQF3	83.87	0.9898	FP, AF	ACE inhibitor
				PA, FP, AF	Dipeptidyl peptidase IV inhibitor
GFFGL	A0A662YXS9	64.52	0.9815	GF, GL, FG, FF	ACE inhibitor
				GL, GF, FF	Dipeptidyl peptidase IV inhibitor
				GF	Dipeptidyl peptidase III inhibitor
FPVF	A0A662YZB8	69.29	0.9749	VF, FP	ACE inhibitor
				FP, PV, VF	Dipeptidyl peptidase IV inhibitor
VGFF	A0A444UCW2	62.26	0.9674	GF, VG, FF	ACE inhibitor
				GF, VG, FF	Dipeptidyl peptidase IV inhibitor
				GF	Dipeptidyl peptidase III inhibitor
GYGF	A0A444UD25	62.26	0.9652	GY, YG, GF	ACE inhibitor
				GF, GY, YG	Dipeptidyl peptidase IV inhibitor
				GF, YG	Dipeptidyl peptidase III inhibitor
				YG	Immunomodulating
MFLL	A0A444UFC8	62.26	0.9636	MF	ACE inhibitor
				LL, FL, MF	Dipeptidyl peptidase IV inhibitor
				FL	Dipeptidyl peptidase III inhibitor
				LL	Stimulating
FLGM	A0A444UQD2	83.87	0.9620	GM, LG	ACE inhibitor
				FL	Dipeptidyl peptidase IV inhibitor
				FL	Dipeptidyl peptidase III inhibitor
GFVF	A0A444UFC8	62.26	0.9611	VF, GF	ACE inhibitor
				GF, VF	Dipeptidyl peptidase IV inhibitor
				GF	Dipeptidyl peptidase III inhibitor
KGMF	A0A662YVD5	72.08	0.9506	MF, GM, KG	ACE inhibitor
				KG, MF	dipeptidyl peptidase IV inhibitor
LPGLF	A0A444UIZ4	83.66	0.9486	LF, PGL, LPG, GL, PG, LP	ACE inhibitor
				LP, GL, PG	Dipeptidyl peptidase IV inhibitor
				GLF	Immunomodulating
				GLF, PG	Regulating
				LL	Stimulating
				PG	Antiamnestic
				PG	Antithrombotic
GLLF	A0A444UQC8	62.26	0.9433	LF, GL, LLF	ACE inhibitor
				LL, GL	Dipeptidyl peptidase IV inhibitor
GFGGL	A0A444U3D5	97.74	0.9319	GF, GL, FG, GG, FGG	ACE inhibitor
				GL, GF, GG	Dipeptidyl peptidase IV inhibitor
				GF	Dipeptidyl peptidase III inhibitor

^a^: From PeptideRanker (http://bioware.ucd.ie/ (accessed on 8 February 2023)). ^b^: From BIOPEP (https://biochemia.uwm.edu.pl/ (accessed on 6 February 2023)).

## Data Availability

Data are available upon request from the authors.
